# Black and White Insanity

**Published:** 1857-01

**Authors:** 


### BLACK AND WHITE INSANITY.

WE do not mean the color of the insanity, but the color of the persons who are deranged. The census states, that in Maine, one out of every fourteen free colored persons was insane. In Virginia, there was at the same time but one insane slave out of every thirteen hundred. We think the reason for this wide difference is soon told. It is the struggle and anxiety for daily bread which eats out the mind of the Northern negro. Slaves have no such anxieties; their lives are merrier than those of their masters; they know that *bread shall be given them and their water shall be sure; and having food and raiment*, they are *therewith content*, measurably. Their religion *makes their yoke easy and their burden light.* Practically, the Southern slave does not feel his bondage as such. There is such a thing as *learning to bear the yoke*, for under a load which would grind the cultivated intellect to powder, a Southern slave, with the aid of his religious faith, will *mount as with the wings of eagles,—will run and not be weary,—will walk and not faint.*

The mass of slaves in our country assent to the religious sentiment either by practice, profession, or proclivity, and have *learned in whatsoever state they are, therewith to be content.* There can be no doubt, that with other aids, the burden of slavery is comparatively light to them. A thousand times have we heard the lively song on the leveé, at New Orleans; it was the song of the slave—the song that helped them to work easy, and they found it out. We never heard a note of music from the hundreds of Irish draymen, always about them, in ten years.

We are not discussing the morality of slavery; we are speaking of it physically; we are stating apparent facts. Their solution is open to discussion. To embody the main point in words, we would express it thus:—

Free negroism in the North, with its surroundings, appears to promote insanity. Southern slavery, with its surroundings, appears to prevent insanity.

We do not say, merely, that Southern slavery does not seem to tend to insanity—that would express only a negation; the truth is positive. *Southern slavery, with its surroundings, prevents insanity*, for it is a statistical fact, that more white people become insane in the South, or are born with various approaches to idiocy, than among slaves in proportion, while as to the whites of New England, there are a thousand lunatics where there is one among the slave population of the South. Many thousands there are in the North who go to bed, nightly, without a full stomach, and in the winter, with the addition of a cold and cheerless apartment; while such things are almost unknown on Southern plantations. If these facts were not known from actual observation, they might find a moral demonstration in the deduction that they were so, because it is to the pecuniary interest of the slave-holder to give that slave food and warmth, as much, and more so, than for the Northerner to afford those things to his cattle, if he wishes to be a thrifty farmer

While these considerations should justly modify Northern sentiment as to the conjectured horrors of slavery, and lead them to take a calm and considerate view of the whole subject, and thus, as we think, open up a speedier way for universal freedom, as far as the great interests of the world require it; they, at the same time, are largely suggestive in the direction of that great subject, human happiness and health.

The effect which the ceaseless struggle against want has on the colored man in New England, manifests itself in the eighty, ninety, and a hundred persons, who die every week in New York of diseases of the brain and nerves. If this ceaseless striving for bread wears out the life of the white man, and drives the black man mad, the very strongest of all earth's considerations force their claims upon us, and urge us, as a means of saving the life, and far more, of the immortal mind, to cultivate in our domestic and social relations, a wise economy and a moderate ambition as to pecuniary condition; for beyond all question, the neglect of this, wrecks the peace and ruins the souls of multitudes.

